# Lived Experience, Concerns, and Support Needs of Adults with Metabolic Dysfunction-Associated Steatotic Liver Disease (MASLD): A Qualitative Study

**DOI:** 10.3390/healthcare14162569

**Published:** 2026-08-17

**Authors:** Sue Shea Wynyard, Lou Atkinson, Chris Kite, Christos Lionis, Harpal S. Randeva, Ioannis Kyrou

**Affiliations:** 1 Warwickshire Institute for the Study of Diabetes, Endocrinology and Metabolism (WISDEM), University Hospitals Coventry and Warwickshire NHS Trust, Coventry CV2 2DX, UK; sue.wynyard@warwick.ac.uk (S.S.W.); c.kite2@wlv.ac.uk (C.K.); 2 Warwick Medical School, University of Warwick, Coventry CV4 7AL, UK; 3 School of Psychology, Clinical & Health Sciences, Aston University, Birmingham B4 7ET, UK; louatkinsonphd@gmail.com; 4 School of Allied and Public Health Sciences, Faculty of Medicine and Health Sciences, University of Wolverhampton, Wolverhampton WV1 1LY, UK; 5 Laboratory of “Health and Science”, School of Medicine, University of Crete, 71003 Heraklion, Greece; lionis@uoc.gr; 6 Department of Psychology, School of Social Sciences and Humanities, University of Limassol, Limassol 3025, Cyprus; 7 Institute for Cardiometabolic Medicine, University Hospitals Coventry and Warwickshire NHS Trust, Coventry CV2 2DX, UK; 8 Discoveries in Life Sciences Research Centre, Coventry University, Coventry CV1 5FB, UK; 9 Aston Medical School, College of Health and Life Sciences, Aston University, Birmingham B4 7ET, UK; 10 College of Health, Psychology and Social Care, University of Derby, Derby DE22 1GB, UK

**Keywords:** metabolic dysfunction-associated steatotic liver disease, MASLD, lived experience, qualitative study, support needs

## Abstract

**Highlights:**

**What are the main findings?**

- The majority of adults with metabolic dysfunction-associated steatotic liver disease (MASLD) who participated in this study reported communication issues at the point of diagnosis including lack of clarity and information. Most had been diagnosed incidentally. Participants highlighted a range of emotions at diagnosis (e.g., confusion, fear, frustration, anxiety, shock, and feeling overwhelmed) and also reported issues related to information available on the internet, feelings of fatigue, and potential stigma.
- The minority of participants reported that they had received follow-up care for MASLD, but most reported concerns regarding lack of follow-up and information. Fears in relation to MASLD progression were raised, particularly in cases where there was a family history of liver disease.

**What are the implications of the main findings?**

- Most participants with MASLD were aware of the importance of lifestyle changes but needed some support to achieve and/or sustain these changes. Thus, a holistic individualized care pathway for MASLD with signposting to reliable information and health education is recommended.
- Patients with MASLD and a family history of liver disease expressed particular concerns regarding disease progression, highlighting this as an additional point that should be taken into consideration to address the needs of these patients regarding issues about MASLD progression and/or longer-term complications.

**Abstract:**

**Background/Objectives**: Metabolic dysfunction-associated steatotic liver disease (MASLD) is caused by excessive fat accumulation in the liver (steatosis) and affects approximately 38% of adults globally. MASLD may progress from simple steatosis to fibrosis and cirrhosis and is closely related to other cardio-metabolic conditions (e.g., obesity and type 2 diabetes), posing a risk factor for cardiovascular disease. Currently, lifestyle modification and weight reduction remain the main initial treatment options. Existing data suggest that there is low awareness among patients regarding MASLD diagnosis and its subsequent management. Therefore, this study aimed to develop a rich understanding of the lived experiences, concerns, and support needs of adults with MASLD. **Methods**: A qualitative design was applied, utilizing semi-structured interviews. Adults living with MASLD were invited to talk about their diagnosis and discuss their lived experiences. Participants were interviewed by telephone or video call, and each interview was transcribed and analyzed with a reflexive thematic analysis approach. **Results**: Twenty-five adults with MASLD (age range: 24–79 years; 40% men) were interviewed. The emergent key themes related to communication and emotions at diagnosis; independently seeking further information; lived experiences post-diagnosis; support needs; and future concerns. Many participants reported receiving the diagnosis incidentally, and a number of issues were raised regarding lack of clarity at the point of diagnosis. Additional concerns included information obtainable via the internet, symptoms, social relationships, stigma, and lifestyle modification. Future anxieties related mainly to fears of disease progression, while support needs were predominantly focused on information and follow-up. **Conclusions**: The concerns and support needs identified by this study highlight key issues/themes that should inform education and support initiatives by relevant healthcare services aiming to improve the lived experiences and holistic management of adults with MASLD.

## 1. Introduction

Metabolic dysfunction-associated steatotic liver disease (MASLD), previously known as non-alcoholic fatty liver disease (NAFLD), is caused by an accumulation of fat in the liver (hepatic steatosis) and affects around 38% of adults worldwide [1], representing the most common condition among chronic liver diseases. MASLD is closely linked to other cardio-metabolic conditions, such as obesity, type 2 diabetes, hypertension, and dyslipidemia [2] and is widely considered to represent the “hepatic manifestation” of metabolic syndrome [3], posing an independent risk factor for cardiovascular disease (CVD) [2,4]. Moreover, systematic review and meta-analysis data also indicate relatively strong relationships between MASLD and common mental health conditions [5].

MASLD may progress from simple steatosis to inflammation and fibrosis, with severe complications over time such as cirrhosis and liver failure [6,7]. Currently, the initial treatment of MASLD still relies predominantly on lifestyle modifications in the form of physical activity and dietary changes [8,9]. However, such lifestyle changes may be problematic for some individuals to achieve and maintain, and the concept of sustained lifestyle modification may be difficult to understand and implement within the context of their own personal circumstances [8,9]. As such, personalized support and lifestyle plans that are appropriate for the individual to follow in the longer term, together with early detection of MASLD, are crucial in order to avoid longer-term disease progression and reduce the risk of CVD [9].

Within this landscape, existing data indicate low awareness among patients regarding the MASLD diagnosis and its management. Indeed, a qualitative study by Deshpande et al. [10] identified that although most participants understood the severity of MASLD, knowledge regarding treatment in the form of exercise was lacking. Furthermore, research data have indicated that confusion and lack of awareness regarding the diagnosis of MASLD may further impede the undertaking of self-management activities and that barriers such as low motivation and comorbidity may contribute to lack of participation in lifestyle modification [11,12]. Overall, studies investigating the level of MASLD awareness have demonstrated low condition awareness even among individuals with high-risk factors, as well as ambiguity surrounding the etiology and diagnosis of the condition [12,13]. Indeed, a recent study involving participants from European primary care settings demonstrated that although a significant number of participants were at risk of MASLD, they were unaware of the condition and associated risk factors. This study highlighted the need to enhance condition awareness for MASLD, especially among high-risk patients, in order to reduce the corresponding risk and promote prevention [14].

In addition to lack of awareness regarding MASLD, existing evidence indicates that a number of factors could interfere with or obstruct the optimum management of MASLD. These include the known associations of MASLD with common mental health problems and factors such as perceived stigma and fatigue, which could place the individual at risk of feelings of social isolation [5,8,9,15,16].

As there are multiple factors that can impact the self-management of MASLD and the individual’s experience of living with the condition, effective communication between healthcare professionals and patients with MASLD is important to increase understanding and better consider the individual’s needs. Within this context, studies have highlighted the significance of communication issues, drawing attention to factors such as trust and mutual appreciation, indicating strong preferences from patients regarding improvements in communication strategies [17,18].

Other studies have explored various aspects in relation to living with MASLD, frequently focusing on single issues relating to awareness, perceived stigma, healthcare experiences, disease knowledge, attitudes, and behaviors. These studies identified a number of issues such as low patient awareness, effects of stigma, limited access to support groups, ambiguity regarding disease etiology, and diagnosis [11,12,16,19]. The current study aimed to add to the relevant literature by providing a broader picture of the lived experience of patients with MASLD, avoiding focusing on a single issue/aspect of living with MASLD. Thus, the present study aimed to capture the entire “journey” of these patients from the point of receiving the diagnosis to living with MASLD post-diagnosis and their thoughts regarding the future in order to identify the key pertinent experiences, thoughts, and feelings, as well as common future concerns and support needs.

Capturing the lived experiences, concerns, and support needs of these patients may help to inform improvements to the holistic management of this prevalent chronic condition.

## 2. Materials and Methods

### 2.1. Study Participants

Adults (>18 years old) with a diagnosis of MASLD/NAFLD were eligible to participate in this study. Although allocating a sample size in qualitative research can be an issue of some debate [20], systematic review data suggest that important information can be obtained with 17 interviews or less [21]. Findings identified from existing research studies among participants with related conditions generally involve sample sizes of around 20 participants [22,23,24,25]. Moreover, in the context of this study, we applied the reflexive thematic analysis approach [26] and the concept of “Information Power” [27]. This concept focusses on “information and richness” of data obtained from participants with specific experiences, rather than on “thematic saturation”. Guided by this concept and previous similar studies, a target sample of 25 participants was considered appropriate for the purpose of this qualitative study, with the option of increasing the sample size if the required “Information Power” was not achieved based on the data from the first 25 recruited participants.

For the recruitment of participants for this study, we aimed to recruit an approximately equal number of male and female participants and also, where possible, to seek diversity in terms of other demographic characteristics, such as age.

The option to volunteer to participate to this study was advertised via a website landing page supported by the British Liver Trust, a leading UK charity organization offering support to individuals with liver disease. Through the British Liver Trust website, interested individuals were able to access a participant information sheet for further information. Those meeting the eligibility criteria (i.e., age > 18 years and with a diagnosis of MASLD or NAFLD) and wishing to participate contacted the research team and were subsequently provided with the consent form. Following informed consent, participants were directed to some short multiple-choice questions regarding diagnosis and demographic details. Participants were then contacted by a member of the research team to arrange a mutually convenient time and date to conduct the interview via Microsoft Teams (Microsoft Corporation, Redmond, WA, USA, v4.3) or by telephone.

### 2.2. Data Collection

Utilizing a topic/question guide (Table 1), which was informed by the pertinent information/topics identified from our previous in-depth literature reviews [5,8,9,15], participants were invited to talk about issues relating to their diagnosis and experience of MASLD, including information provided and follow-up received, as well as about their lived experiences of MASLD (e.g., symptoms, emotions, effects on social relationships, and support needs). Each interview lasted approximately 30–40 min, with an average duration of 30 min. Participant responses were audio or video recorded, and the recordings were subsequently transcribed. Any personal details that could potentially identify the participants were deleted to protect anonymity, and transcripts were assigned a unique code to maintain confidentiality.

Where any emotional distress was potentially present during the interviews, the interviewer demonstrated empathy and offered the option to take a break, move onto a different topic, or end the interview. This approach was also outlined in the participant information sheet of the study. In addition, the interviewer recommended that the participant contact the nurse-led helpline of the British Liver Trust and/or other similar resources that they could access via their GP and/or relevant healthcare provider.

This study adheres to the Standards for Reporting Qualitative Research (SRQR) [28], (Supplementary Table S1).

### 2.3. Data Analysis

A reflexive thematic analysis procedure was adopted, which represents a useful approach for identifying common themes and sub-themes that might emerge [29]. Thematic analysis of transcripts was conducted by the first author (SSW), who has a background in health psychology and chronic disorders with a focus on MASLD and its association with mental health. The analysis was performed manually, without the use of any qualitative analysis software.

**Figure 1:**
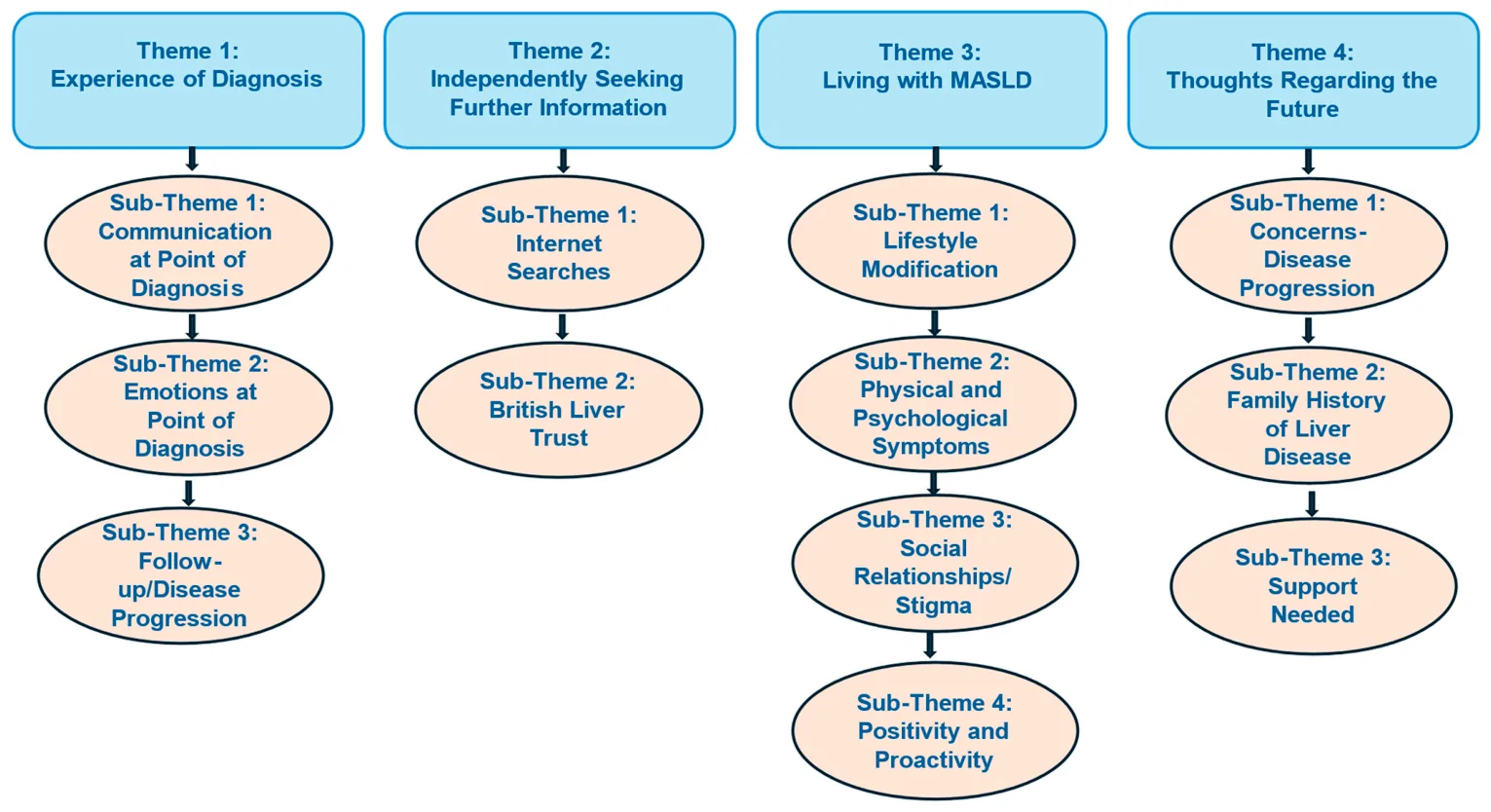
Emergent themes and sub-themes.

A reflexive approach was utilized, emphasizing reflection and subjectivity to capture the in-depth meaning of the data [26]. The epistemological orientation underpinning the reflexive thematic analysis utilized within this study is interpretive and focuses on the experiences and perceptions of each individual participant rather than knowledge that is objectively discovered. Furthermore, the approach embraces the researcher’s subjectivity as an important resource. A six-phase procedure was followed, consisting of the following: (1) Familiarization with the data: The audio recordings were listened to at least twice, while the transcripts were also read at least twice. This process allowed for full immersion and absorption of the data in order to ensure familiarity with each data item and with the entire dataset. (2) Generating initial codes: The data were systematically studied, on a line-by-line basis, in order to break the data down and identify meaningful sections. Labels were then assigned to the identified sections of data to capture individual concepts. At this stage, a total of 32 codes were identified. (3) Searching for themes: The codes were studied and organized to identify a broader meaning and connections between them. Codes relating to a shared concept were collated and assigned an initial theme to generate an initial broader theme. (4) Reviewing themes: The initial generated themes were cross-checked to ensure that the data within them belonged together and related to the full dataset. Where appropriate, initial themes were either joined together or split to form a new theme. (5) Defining and naming themes: The focus of each theme was analyzed and assigned a name to capture the underlying concept and ensure applicability to the research question. (6) Writing up the report [30]. The procedure resulted in four themes and 12 sub-themes (Figure 1).

Throughout the reflexive process, SSW was consistently aware of her position and possible influence on the interpretation of the data. The transcripts were re-read several times, and a reflexive diary was introduced in order to record responses to the data. Lists of codes and theme definitions were also kept as part of an audit trail. The analysis and results were subsequently shared and discussed with all co-authors.

### 2.4. Ethical Approval

Ethical approval for this study was obtained in January 2025 from the Health Professions Ethics Committee (Ref: 1124CKUOWHEA) at the University of Wolverhampton, UK.

## 3. Results

### 3.1. Demographic and Clinical Characteristics

A total of 25 adults were enrolled in the study between June and December 2025, including 10 (40%) male and 15 (60%) female participants, with a mean age of 57 years (age range: 24–79 years). Most participants (n = 16; 64%) reported receiving their diagnosis based on a liver ultrasound or Fibroscan, while additional diagnostic examinations by computed tomography, magnetic resonance imaging, or both were, respectively, reported by five (20%), two (8%), and one (4%) of the participants. Only one (4%) reported a diagnosis via other (not specified) means. Table 2 summarizes the selected pertinent demographic and clinical characteristics that were reported by the study participants.

### 3.2. Emergent Themes and Sub-Themes

During the semi-structured interviews, participants shared a number of experiences, resulting in the generation of the themes and sub-themes shown in Figure 1. The themes and sub-themes are further outlined in the following sections, along with corresponding illustrative quotes.

### 3.3. Theme 1: Experience of Diagnosis

This theme focusses on participants’ experiences both at the point of and beyond the initial diagnosis. Covering factors such as communication, emotions, follow-up, and disease progression, this theme helps to gain a perspective on the participants’ lived experience from the beginning of their personal journey regarding their MASLD diagnosis.

#### 3.3.1. Communication at the Point of Diagnosis

The majority of participants referred to communication issues with physicians and healthcare professionals (HCPs), and some expressed disappointment regarding the way in which the diagnosis had been delivered. This was particularly pertinent in instances where the diagnosis had been relayed via text message or telephone call, or where the diagnosis had only been identified by checking the NHS App. The importance of face-to-face contact was raised, with one participant stating the following:

*“… I’ve never actually seen anybody… I personally think face to face—I know it’s hard these days with the demands, but when you’re having that diagnosis, I think it’s quite important to have that person tell you and then you can gauge how serious it is or, you know, you can ask questions. It’s just different over the phone…”.*

*(QP04)*

While time and resources may impede the opportunity to engage in face-to-face contact, the effects of receiving a diagnosis via other means can lead to uncertainty and further distress to the individual. Moreover, being able to ask questions and to have the opportunity to ascertain the seriousness of the diagnosis is emphasized in cases where no direct contact has taken place, thus highlighting the importance of a more personalized approach at the point of initial diagnosis.

Problems in relation to late diagnosis were also shared by some participants, whereby they had not received the diagnosis until several weeks after testing. One participant mentioned the following:

*“…they kind of didn’t really tell me what they’d seen until much later and then told me oh yes, fatty liver…”.*

*(QP013)*

Another stated the following:

*“…they found fatty liver and an enlarged spleen. But I wasn’t informed that they found that…it was only when I got a GP appointment about a month later, and she said ‘oh yes, you’ve got a fatty liver’…”.*

*(QP020)*

In a number of cases, the MASLD diagnosis represented an incidental finding, wherein the participant had undergone routine testing for an existing disorder, or they had reported to their physician with symptoms related to other conditions.

One participant commented the following:

*“…so, it was just one of those findings that was incidental because I had gone for an ultrasound scan for something else and that just showed up…”.*

*(QP07)*

The implications of late diagnosis and incidental findings further warrant the need for a full explanation at the point of diagnosis, given that the individual is likely to feel confused and shocked. In cases where tests had been performed but the diagnosis had not been relayed until some weeks later, the individual may have assumed that the lack of an initial diagnosis indicated that no further health issues were evident. As such, valuable time may have been lost in terms of taking action or engaging in some form of lifestyle modification. The delay in initial diagnosis perhaps reflects communication issues across different aspects of the medical system, while the issue of “incidental” findings suggests that further studies should explore the optimal screening approaches for individuals likely to be at risk of MASLD.

Furthermore, participants reported confusion regarding lack of clarity surrounding the diagnosis together with frustration in relation to the perceived lack of concern expressed by their general practitioners (GPs) or HCPs. Participants generally felt that GPs and HCPs were somewhat vague in their approach and that they were “not taking the condition seriously” and were potentially “playing down” the seriousness of MASLD. Participants felt frustrated by the indications that MASLD was a common condition that could only be treated through lifestyle changes, with one describing the physician’s attitude as *“…flippant—not taking it particularly seriously…” (QP024).*

One participant said that when they inquired about the cause of MASLD, the physician responded by saying, *“…well, it’s just unlucky, really…” (QP04).* Another participant noted that she was told the following:

*“…don’t worry, it’s just a fatty liver, and I promise you now…most of the population probably is walking around with a fatty liver…”.*

*(QP02)*

When asked about the information received at the point of diagnosis, most participants explained that they had received little or no information, pointing to the need for bonafide information leaflets or signposting to such.

One participant reflected on his amazement upon asking if there was any information or literature that he could be referred to:

*“…I asked about is there any information, is there any literature that you can send out or that you can possibly signpost me to, and I was recommended to Google it…”.*

*(QP01)*

Other responses included the following: *“…I’ve never been given any literature on it or anything like that…” (QP011),* and *“…She just told me what I’ve got…basically it was just got this, change your lifestyle, lose weight…” (QP014).*

The vague approach to the MASLD diagnosis and lack of information provided at this point represented recurring themes, with one participant stating the following: *“…knowledge is key…you can’t expect people to manage it if they haven’t got the knowledge…” (QP010).* Such lack of clarity and information further highlights the need for effective communication to aid in the elimination of further psychological distress and confusion. Furthermore, insufficient information may result in decreased motivation to engage in the lifestyle modifications required to manage the condition.

However, despite the fact that many participants reported a lack of information, one participant noted that the information received was good, and two participants stated that they had been provided with information leaflets. In addition, two had been informed about the work of the British Liver Trust. This perhaps demonstrates a lack of consistency across the healthcare system, and as such, promoting examples of good practice could prove crucial.

Seeking clarification and further information by attempting to secure a GP appointment was also raised, with one participant reporting the following:

*“…trying to even get through to the reception staff is unbelievable. The telephone line is open at 9:00 and at 9:00 o’clock on the dot you ring, and you get beep, beep beep…then about 90 s later, a message comes up saying all the appointments for today are now filled…”.*

*(QP017)*

Another participant expressed dissatisfaction regarding being unable to obtain an appointment with a regular GP, stating the following:

*“…To get an appointment with a regular doctor…this thing was all done by a locum, you know, with the best will in the world, they are keyboard warriors and they’re working off the screen…”.*

*(QP011)*

Such experiences further underscore the importance of effective communication between the individual and the HCP responsible for their care in order to provide opportunities for discussion in relation to the diagnosis and to discuss an appropriate care pathway. Being unable to secure a GP appointment likely leads to further frustration, contributing to the concerns relating to the MASLD diagnosis.

#### 3.3.2. Emotions Experienced at the Point of Diagnosis

When asked about their thoughts and feelings at the point of diagnosis, participants expressed a range of emotions. These emotions included confusion, anger, frustration, anxiety, fear, shock, and feeling overwhelmed and were shared by the majority of participants. One participant stated, *“…I was really shocked. I was really upset. I’m scared. Yeah, I was scared…” (QP025),* while another reported, *“…when I first received the diagnosis, I was very scared, and I was unable to sleep at night…” (QP09).*

Furthermore, one participant drew attention to the potential distress of not understanding the diagnosis:

*“…I sometimes think they forget that after they’ve given the diagnosis to somebody, that person goes away, they don’t understand all the medical implications of it….and I think the doctors forget that somebody is maybe sitting at home in distress, without any real help…”.*

*(QP017)*

The above is somewhat alarming, suggesting the possibility of perceived isolation regarding the diagnosis itself, which may result from confusion regarding the meaning of the diagnosis in terms of the individual’s personal circumstances or medical history. The range of emotions expressed by participants highlights the importance of addressing such concerns to avoid feelings of isolation and further emotional disturbances in the longer term that may result in a lack of motivation to manage the condition.

However, it is of note that one participant described the diagnosis as a “wake-up call”, while another reported feeling a sense of relief that the diagnosis was not something more serious.

#### 3.3.3. Follow-Up/Disease Progression After Diagnosis

Most participants reported disappointment regarding the lack of follow-up and were frustrated in cases where they were actively pursuing further testing and follow-up but had been unsuccessful in obtaining this. One participant remarked,

*“…I did say so, what’s the plan now? And I was told, well, there’s nothing…you just got it…” (QP011),* whilst another said: *“…there’s no actual follow up…I was told that they may want to do blood tests in the future, but I’ve not heard anything from my doctors…”.*

*(QP012)*

A minority of participants reported that they were receiving follow-up care, with two of them reporting satisfaction with this. One participant drew attention to regional differences and described being fortunate to reside in an area where provision for healthcare was exceptionally good:

*“…the local hospitals, they’ve been excellent as well… I’ve had my six-monthly checkups, very regularly and discussions each time face to face with the liver nurse at the hospital. And they’ve been very happy to supply me with not just the verbal information but written and also the scores from my blood tests, because I know that in some areas, they have problems with getting those…they’re very good in this area…”.*

*(QP022)*

However, the majority of participants did not appear to be receiving any follow-up care or testing.

A further issue of concern relates to disease progression, as it seems that in a few cases, while participants may have initially been informed of an accumulation of liver fat, their condition had since advanced, which was naturally daunting and worrying for them. One participant commented,

*“…it wasn’t until last November that the doctor said ‘Oh, you should have had this all explained to you as this is quite serious cause you’ve now increased to a liver stiffness of 11 whereas in 2020 it was only 6′. So, it’s obviously progressed quite a lot… I was quite shocked when… Oh my God you know…what am I gonna do sort of thing…”.*

*(QP04)*

The above accentuates the importance of monitoring the condition, both in terms of ensuring that the individual is kept informed and to prevent disease progression. Without sufficient follow-up and information, there is again a possible risk that the individual may develop further emotional distress or may become less motivated in terms of managing their condition. The example of good practice outlined by participant QP022 again draws attention to inconsistencies across the healthcare system.

### 3.4. Theme 2: Independently Seeking Further Information

This theme addresses the methods utilized by participants in order to obtain further information regarding their condition.

#### 3.4.1. Internet Searches

When participants were asked if they had located further information regarding their condition, the majority stated that they had searched online but were disappointed with the information available on the internet. Most participants expressed distrust in this method of attempting to find reliable facts and advice. Many felt that information located via the internet was too generic or that it either underplayed the condition or encouraged further fear and confusion. One participant commented, *“…but that’s it…other than Googling it…you hope for the best and you read the worst…” (QP019),* whilst another stated: *“…going through the wrong things and looking on Google and stuff, which was no good to me whatsoever…that’s a disaster, isn’t it? Going down that road…according to Google, I’m dying next week…” (QP022)*.

However, participants who had located and utilized the British Liver Trust were very satisfied with this method of obtaining further advice and information, as detailed in the next section.

#### 3.4.2. British Liver Trust as a Source of MASLD Information

Participants reported being very satisfied with the information available on the website of the British Liver Trust. Many participants reported joining the British Liver Trust support groups, signing up for their newsletter, utilizing the information on the website pages, and/or calling the nurse helpline for assistance and advice. One participant told us, *“…I then came across the BLT, and I phoned their helpline and the nurses were great. They helped me put things into perspective…” (QP09).*

Participants described the information available via the British Liver Trust as reliable, evidence-based, and encouraging, as it helped them to raise specific questions with HCPs. However, many participants expressed dissapointment with the fact that they had had to spend time searching through many unreliable websites prior to finding the webpage of the British Liver Trust. Participants felt that their GP or HCP should have referred them to this resource at the point at which they were diagnosed:

*“…Yeah, I think it’s something that GP’s should advertise more in their surgeries. But then who gets to go into a doctor’s surgery?” (QP024),* and

*“…if our hospital doesn’t have the money to print info, you know, or pay the British Liver Trust for their good info, there’s no excuse for people not to be given A4 sheets of paper with, you know, website addresses…”.*

*(QP023)*

The above further underscores the importance of being provided with reliable information at the point of diagnosis. It is perhaps not surprising that most participants had turned to the internet in order to obtain more details about their condition and how to manage it. However, many participants were disatissfied with the information available online, due to this being either too generic in nature or inducing further fear and concerns regarding disease progression. Participants were happy with the information provided by the the British Liver Trust, which again reinforces the importance of effective communication to avoid further psychological or physical complications in the longer term.

### 3.5. Theme 3: Living with MASLD

This theme explores participants’ lived experiences post-diagnosis, focusing on factors such as lifestyle modification, physical and psychological symptoms, social relationships, and perceived stigma. The theme further describes participants expressing positive attitudes or taking a proactive stance towards living with MASLD.

#### 3.5.1. Lifestyle Modification

While most participants understood the importance of lifestyle modification, many considered this instruction to be somewhat vague, and some felt that they needed more information relevant to their specific journey and circumstances. The majority felt unhappy with simply being told to change their lifestyle, with one stating, *“…all that they were telling me was change your lifestyle, change your lifestyle, change your lifestyle…” (QP015).* Many had already made efforts to engage in dietary modifications and increased physical activity, with one participant reporting, *“…I have put the brakes on massively and I’ve changed my lifestyle and consequently I’m seeing the results…” (QP021).*

However, several participants were unsure as to what further changes were expected in order to improve their condition, particularly in cases where they were already at a healthy weight. Indeed, one participant mentioned, *“…they have me down as obese and I’m certainly not that…*” *(QP04)*. In some cases, participants reported that weight had always been an issue for them, and therefore trying to lose weight was somewhat challenging: *“…I am overweight, but that’s been the case for donkey’s years, so it’s quite hard to do anything other than shift the odd half stone here and there…” (QP011).*

Some participants expressed a need for support to aid them in lifestyle modification procedures. One participant stated, *“…They just said you’ll have to diet…I know that already, but I want a bit of help…So I’ve not got any help off them…” (QP016).*

Furthermore, one participant mentioned the benefits of being referred to a weight management programme: *“…the next thing that they did is they put me in a weight management programme…I did this weight programme, I’m doing better…” (QP06).*

In some cases, participants reported current barriers to physical activity participation resulting from injury, surgery, or other health conditions or symptoms including breathlessness. In addition, personal family issues, trauma, or caring responsibilities were a priority to the individual participant. Indeed, one participant told us the following:

*“…I used to be far more active than now, but then I ran into an episode of sciatica and sort of because of that, you know, the sort of muscle weakness on that side ended up falling down…”.*

*(QP023)*

Another mentioned the following:

*“…so a lot of my time caring for my son… …well, my son has got… so yeah, and that’s that takes up a lot of time…”.*

*(QP020)*

These comments further highlight the importance of considering the individual circumstances of the individual. Many people find lifestyle modification challenging, particularly when no clear information is available. There are several factors that may represent barriers to selfcare, which if not addressed could result in feelings of guilt or self-blame. Furthermore, issues such as self-efficacy in relation to lifestyle changes or lack of health education regarding MASLD may have an impact on the extent to which such changes are achievable. Thus, monitoring the individual and providing support could help to overcome such challenges.

#### 3.5.2. Physical and Psychological Symptoms

Participants were invited to share information regarding physical or psychological symptoms that they believed to be related to MASLD. However, due to the presence of other health conditions, most participants were unsure as to whether the physical symptoms that they experienced were due to MASLD or what symptoms they should expect regarding the condition.

One participant noted the following:

*“…it’s difficult to say because having had a gallbladder removed…I’ve always experienced some discomfort under my ribs on the right-hand side. So, I wasn’t sure whether or not that was to do with it or not…”.*

*(QP024)*

Other participants reported abdominal pain, discomfort, or aching on the lower right side of the abdomen, and two participants mentioned feeling “itchy”. In addition, some participants reported that they had been previosly diagnosed with sleep apnea, and the majority of participants referred to feelings of fatigue and tiredness:

*“…I’ve got sleep apnea as well, so…I don’t sleep…It’s just a vicious cycle of no sleep, no energy, no motivation…” (QP014),* and

*“… I’m wiped out completely. I wake up tired. I could fall asleep now, you know. It’s just…it’s complete fatigue…” (QP025),* as well as

*“…I feel a lot tired. Depending on the day, it does affect my ability to do exercise. I feel like I just want to lay down on the sofa during the whole day…”.*

*(QP06)*

With regard to psychological symptoms, some participants mentioned ongoing emotions regarding their condition, while others reported a history of anxiety or depression in general, not specifically related to the diagnosis of MASLD.

Due to the lack of information regarding their condition, participants were unsure regarding related symptoms. However, fatigue and sleep disturbances represented a recurring theme, which is likely to impede physical activity levels and emotional health.

#### 3.5.3. Social Relationships and Perceived Stigma

Participants held mixed views on whether the condition affected their social relationships. Some did not feel that the diagnosis of MASLD had particularly affected their social relationships, while others mentioned concerns regarding eating out, avoiding alcohol, and socially withdrawing due to fatigue or mobility issues.

One participant mentioned the following:

*“…No, I have not avoided… but I have been aware like oh God, going there… what should I do? Should I not eat? Should I eat? So, I suppose it has been on my mind, but I’ve not seen it as a problem…”.*

*(QP025)*

An additional participant shared the following:

*“…. I noticed I was withdrawing…my friend would ring me, and I would not answer the phone…I’m just not feeling up to it, so I would avoid people, not in a horrible way, but just in…I have to protect my own energy way…”.*

*(QP02)*

Another said the following (within the context of social pressure):

*“…I’m not doing parties and all because I’m… I cannot go… I cannot eat, I cannot drink…people come with recipes for you and then I want to avoid that……people say, don’t worry, fatty liver is normal, everyone has it…it’s very common…”.*

*(QP03)*

Almost all participants referred to an assumed relationship between alcohol and liver conditions. Most participants reported only being occasional drinkers prior to diagnosis, and those who had consumed more alcohol had sinced stopped completely:

*“…I mean, I don’t drink and I was never a big drinker, but I used to have the occasional one. I suppose you go out, you sort of think ooh… I wouldn’t go out with people that drink a lot because I would just be sitting there…” (QP04),* and

*“…I just can’t have a drink, you know, have to have a non-alcoholic drink, so…and even then, I have to watch the sugar contents because a lot of drinks have so much sugar, so I tend to drink sparkling water or something like that…”.*

*(QP05)*

Furthermore, perceived stigma appeared to be an issue, with some participants sharing their concerns that they would be judged in public if discussing their condition due to the association of liver disease and alcohol. One participant shared the following:

*“Stigma is the other issue…they will think I’m a heavy drinker and I hardly ever drink…”.*

*(QP09)*

Another said,

*“…people just look at you and think you’re an alcoholic or whatever. You know, that you’ve brought it on yourself, and there is that judgement, you can see it…”.*

*(QP02)*

Another participant referred to weight issues as a potential factor with regard to perceived stigmatization:

*“…I do feel there’s quite, there’s a lot of negativity it it’s, you know, it’s almost a diagnosis that people just think, wow, you should stop eating so much, you know. It’s a bit of…I almost feel slightly embarrassed about telling people because, you know, I do feel that they look at you and think, well, this is your own fault…”.*

*(QP020)*

Avoidance of social situations either because of social pressure to eat and drink or because of perceived stigmatization surrounding liver disease further highlights the importance of accurate information and disease awareness regarding MASLD. Social withdrawal represents an issue of concern owing to the risk of social isolation or loneliness, which could have a profound effect on quality of life and may further exacerbate mental and physical health in the future. Perceived stigma and social pressure perhaps reflect lack of public awareness regarding MASLD, and as such, raising awareness could prove paramount to easing social discomfort.

#### 3.5.4. Positivity and Proactivity

Some participants emphasized the importance of staying positive and avoiding becoming depressed regarding the condition. One participant mentioned the benefits of attending a well-being café, held at a local surgery:

*“…I try to be positive, yeah…. well, you know, every Wednesday, in the surgery, we have the well-being café, 11–1 every Wednesday—we have a laugh… it’s for patients in the surgery so…and we sit around and we talk about our conditions, mostly chit chats and a bit of music and a bit of a sing song.”.*

*(QP05)*

Other participants had taken a proactive stance by actively seeking/insisting on further testing, paying privately for scans, or attending facilities offered by the “Love your Liver” roadshows of the British Liver Trust. One participant mentioned that the British Liver Trust support groups emphasize the importance of asking for information, rather than waiting for it to be provided. Some participants were also engaging in activities such as fun runs:

*“…I do fun runs……we have a lovely community of people that have had liver transplants or going through things and they start to kind of help like promote the positive things which I think is good…”.*

*(QP01)*

Others were engaged in community projects to advocate and raise awareness regarding liver health:

*“…and ever since I’ve been campaigning and raising awareness and wanting to do as much as I can in my area, because there’s just nothing for people with liver disease…”.*

*(QP02)*

Positive thinking and proactivity may represent crucial elements with regard to enhancing social relationships, raising awareness, and feeling “involved” by way of shared experiences.

### 3.6. Theme 4: Thoughts Regarding the Future

#### 3.6.1. Concerns Regarding Disease Progression

Participants expressed concerns mainly relating to the potential worsening of their condition and disease progression. They felt it was important to know if there is anything further that they should be doing to avoid disease progression. One participant referred to the condition as *“…a ticking time bomb if there aren’t options to do something about it…” (QP023)*. Concerns were also raised regarding fears related to a lack of information, with participants wishing to know whether the condition had reversed and the liver had recovered or if there is something more they should be doing for themselves.

Participants commented,

*“…just worrying about… is the next visit to the doctor going to be the one where we say, I’m sorry, but things have progressed…” (QP019),* and

*“And then you don’t know…how do you know when it’s gone, if it’s gone?”.*

*(QP025)*

Overall, disease progression represented the main concern raised by participants in relation to future thoughts regarding their condition. Lack of monitoring and follow-up is likely to enhance concerns regarding the future and disease progression, thus potentially adding to emotional distress.

#### 3.6.2. Family History of Liver Disease

Five participants reported a family history of liver cancer as an issue of concern and a contributing factor regarding worries about disease progression and potential genetic factors. One of these participants expressed disappointment that annual reviews had not been conducted:

*“…my mum died of liver cancer…It is disappointing, you know, especially when I found out that I should have been reviewed yearly, and I’ve not been… I would have thought maybe it would have lit up on my notes or something…”.*

*(QP016)*

Another mentioned concern following the MASLD diagnosis, due to family history:

*“…well, obviously I was concerned because there is liver cancer and pancreatic cancer in my family…”.*

*(QP024)*

Where a family history of liver disease is evident, there are typically enhanced concerns regarding disease prognosis. This highlights the potential importance of further studies exploring and evaluating the possible benefits of early screening for at-risk individuals. 

#### 3.6.3. Support Needed

As with the points raised regarding the communication/support at the point of diagnosis, many participants felt that they would benefit from further information regarding their precise diagnosis, together with follow-up and appointments with their GP to either reassure them that their condition was not worsening or to inform them of any improvements:

*“…I don’t understand why they put these tests results on the NHS app where the patient can see them, and yet they do not follow up on this…I could not see my GP…”.*

*(QP09)*

Likewise, some participants referred to difficulties getting GP appointments and lack of a clear pathway regarding monitoring of their condition. However, two participants reported that their follow-up was good, and some had been invited for further blood tests or specialist appointments at a later date. One participant mentioned that although she was receiving annual follow-ups, these were often moved to a later date and had only ever been by telephone and not in person.

The required support outlined by participants indicates the importance of introducing clear treatment pathways, with the provision of information at the point of diagnosis through to close monitoring and follow-up of the condition, taking the individual’s personal circumstances into consideration.

## 4. Discussion

The present qualitative study offers detailed insight into the personal journey, perspectives, and lived experience of adults living with MASLD in the UK, from the point of diagnosis to thoughts regarding the future.

### 4.1. Participant Experience at the Point of Diagnosis

Being diagnosed with a chronic condition can be a bewildering experience, and the diagnosis of MASLD has frequently been associated with reduced health-related quality of life and impaired patient-reported outcomes [31,32,33]. In the context of MASLD, the often asymptomatic nature of the disease, particularly in the early stages, further results in a coincidental diagnosis. Indeed, many of the participants in the current study reported that their diagnosis was “incidental”, resulting from routine follow-ups for other existing conditions or symptoms related to a different disorder. This finding is consistent with other qualitative studies [12,16] that have sought to investigate patient experiences in relation to MASLD. The timing of the diagnosis represents an important issue, since early detection and management of MASLD can help to decrease the risk of related complications and comorbidities such as CVD [34]. As such, screening among potential at-risk patients is a factor that could be explored and evaluated in future studies. With the “incidental” aspect of the diagnosis in mind, it is perhaps unsurprising that most of the participants in our study described a range of emotions upon receiving the diagnosis, with “shock” being very commonly reported as the initial emotional response. However, it would appear that in many cases, emotional responses were further exacerbated by the fact that many participants were disappointed by the lack of concern and clarity with which the diagnosis had been presented to them and/or the method by which it had been communicated. Problems regarding communication, MASLD diagnosis, and the manner in which the diagnosis is delivered have been reported in other studies [12,16,19]. Furthermore, participants reported further feelings of frustration due to lack of receiving adequate information, which is consistent with reports from other studies [12,16]. It is also interesting to note that three of our participants came from a medical background themselves but still faced similar issues in terms of concerns, information, and monitoring of their condition.

Since the primary healthcare setting is frequently where the MASLD diagnosis takes place in the UK, the limited information and absence of concern may reflect issues such as physician lack of knowledge and confidence when addressing such a diagnosis [35], thus having an impact on effective communication. A recent European survey identified that lack of awareness, together with limited evidence-based care pathways, and country-specific clinical guidelines were likely to be contributing factors relating to the prevention of optimal care by physicians [36]. This further highlights the need for targeted physician education and training [36]. A further nationwide survey conducted in Canada among primary care physicians revealed a lack of knowledge about the condition, despite an understanding of MASLD as an important health problem; thus, further education was suggested in order to address this knowledge gap [37]. Taking these factors into account and attempting to address barriers to patient healthcare, a European team of experts was recently convened in order to develop a set of evidence-based recommendations for primary care settings [38]. It is envisaged that dissemination of such recommendations could contribute to improvements in the care of patients with MASLD within the primary health-care setting [38].

Furthermore, although the primary healthcare setting may be pivotal to the diagnosis and management of MASLD, since the COVID-19 pandemic, there has been a reduction in the number of face-to-face appointments [9], increasing the frustration reported by participants in the present study. A recent systematic review exploring patient preferences regarding UK general practice further revealed that patients would like a choice regarding the mode of consultation, together with more information regarding how to access general practice [39]. However, it should be noted that there is a shortage of GPs within the UK, with data suggesting that there is currently only one GP for every 2220 patients [40], and thus in-person appointments and annual follow-ups are likely to prove difficult.

However, in some cases, the condition had progressed since the initial diagnosis, again perhaps reflecting a lack of knowledge regarding MASLD within the primary healthcare setting. Moreover, it is of note that in some cases, it might be relevant to refer a patient to a secondary care setting, particularly in the case of high-risk individuals and those at high CVD risk, in order to address specific care needs, as reflected in the Clinical Care Pathway developed by Kanwal et al. [41].

It is of further interest to note that the issue of regional differences was raised by one participant in our study, who described the care received and the follow-up monitoring of his condition within the secondary care setting as “excellent”. However, this was an exception among the participants in this study. Indeed, a recent survey aimed at exploring the extent to which UK health authorities and clinical commissioning groups engaged with community chronic liver disease management identified variations across the surveyed UK regions. The study revealed predominantly low levels of engagement, with only 20% of health authorities and clinical commissioning groups identifying a specific individual with responsibility for liver disease [42]. These authors suggest that the findings from that survey should be used as an incentive to implement change and draw attention to the improvements needed regarding primary care management of chronic liver conditions, particularly given their increasing prevalence.

Lack of information provided at the point of diagnosis was a recurring theme among the majority of participants interviewed, and most were not familiar with the condition. Participants were keen to learn more about MASLD, but they felt that they were not provided with sufficient information and that information available on the internet was unreliable or too generic. It is plausible that health literacy issues may also be a contributing factor in this context, and this point should be further explored by future studies that focus specifically on the level of understanding of these patients and their health literacy regarding the etiology, pathological mechanisms, prognostic trajectories, and treatments of MASLD.

Participants described having to search online for more information about their condition after their diagnosis. However, they expressed disappointment and concerns regarding the information available online and the accuracy of the websites identified. Indeed, the issue of online and AI-generated health information has recently been raised by the UK Royal College of General Practitioners [43], suggesting that while it may be constructive for patients to take an interest in their own health, there are potential safety issues regarding information that lacks context, reassurance, and empathy. Furthermore, such online information may be lacking clinical reliability and evidence standards.

Most participants eventually managed to locate the British Liver Trust website, which they found very helpful and informative. Nevertheless, participants raised further concerns owing to the fact that it had taken some time to learn of the existence of this website, and some felt that they should have been signposted to this at the point of diagnosis, especially given the importance and usefulness of such a resource.

### 4.2. Participant Experience Post-Diagnosis

Most of the participants in our study were aware of the importance of lifestyle changes but felt that the basic instruction to modify one’s lifestyle was insufficient to help them to achieve this. Many reported that they needed guidance and support specific to their personal circumstances. The issue of basic advice to “lose weight” was also reflected in a study by Jensen-LeBlanc et al. [16], whereby participants felt that the instruction to simply “lose weight” was vague and oversimplified.

Within the current study, although many participants were attempting to engage in lifestyle changes, certain barriers were reported by some individuals, including mobility issues due to injury or surgery, family issues, lack of time, and fatigue. While lack of time and fatigue represent common barriers with regard to physical activity [44], it is possible that such barriers are likely to be exacerbated among patients living with MASLD.

Likewise, findings from a study by Deshpande et al. [10] report that some of the key barriers to physical activity included lack of energy, injury, and fatigue. These authors suggest that factors such as fatigue and low energy levels could be related to reduced cardiorespiratory fitness, resulting in physical activity becoming more of a challenge [10]. In some cases, participants in our study reported that efforts to lose weight were unsuccessful, potentially impacting on the motivation to continue. Low motivation was also a factor reported in a study by Tincopa et al. [12], wherein the authors suggest that treatment recommendations should be personalized to address the barriers and facilitators experienced by the individual [12].

The majority of the participants in our study reported the presence of additional health conditions. Thus, when asked if they experienced physical symptoms related to MASLD, they were unable to clearly ascertain whether any discomfort or pain that they currently encountered was related to MASLD. Furthermore, participants noted that it was difficult to judge, because they had not been informed of what symptoms they should expect to experience directly in relation to MASLD. The most recurring reported symptom was related to fatigue, with many participants stating that they felt extremely tired, or lacking in energy. This is in line with evidence indicating that the most commonly reported symptom among patients with MASLD relates to fatigue and sleep disturbances [8,45]. MASLD, particularly during the early stages, is generally asymptomatic, and, due to this lack of obvious symptoms, the condition is often referred to as a “silent epidemic” [46]. However, symptoms such as fatigue and sleep disturbance appear to represent issues commonly reported among patients with MASLD [32,47,48]. Moreover, the consequences of fatigue may result in impaired quality of life and depression [47], as well as poorer physical health outcomes [32].

Regarding psychological symptoms believed to be associated with having MASLD, some participants reported ongoing emotions regarding the diagnosis, while others reported they did not think that the condition itself directly affected them psychologically. Of note, in some cases, participants reported a history of anxiety or depression prior to the MASLD diagnosis. While it is not within the scope of this study to identify relationships between MASLD and mental health, existing data suggest that certain underlying mechanisms may be responsible for a bidirectional association between MASLD and common mental health problems [5].

Although participants had mixed views on whether the MASLD diagnosis affected their social relationships, fatigue was raised as a factor that sometimes prevented them from engaging in social events. Concerns about eating out, not knowing what to eat, or social pressure to consume unsuitable foods were also mentioned, together with the perception that other people perhaps do not really understand the potential seriousness of having “fatty liver”. Moreover, alcohol consumption represented a commonly recurring theme. A small number of participants reported having consumed alcohol in the past, and some reported that they had only been occasional drinkers. However, the majority reported that they no longer consumed any alcohol or had not consumed alcohol for a considerable length of time. Most participants referred to the known relationship of alcohol with liver disease and felt that socializing/social drinking and having even one drink might jeopardize their future health.

Worryingly, the concept of perceived stigma also emerged, as some participants felt that they would be judged by others regarding their diagnosis, even though they were not drinking alcohol. Additionally, weight was mentioned as a feature that may lead to other people assuming that the diagnosis of MASLD was the individual’s “own fault”. The issue of perceived stigma has been raised in other studies [8,16,49], including in a recent qualitative study by Jensen-LeBlanc et al. [16]. That study identified a profound effect of stigma among individuals with MASLD at a number of levels, including socially, emotionally, and in terms of their experiences with the healthcare system. Likewise, Carol et al. [49] report the frequency with which perceived stigmatization may occur among MASLD patients and the effects of this concept on quality of life, regardless of disease stage.

Notably, in 2023, consensus was reached to change the name of the condition from NAFLD to MASLD [50], with one of the reasons for this change being to reduce stigmatization potentially associated with the term NAFLD. However, when the name change was mentioned to participants during the current study, many were unaware that such a change in nomenclature had taken place or had not been informed of this at the point of diagnosis. Some participants responded with comments such as “*that’s a bit of a mouthful* (MASLD)”, others expressed a preference for the original name (NAFLD), and some preferred to simply use the term “fatty liver”. Likewise, a qualitative study aiming to explore nomenclature preferences among patients with MASLD identified that 17 of the 25 participants interviewed expressed a preference for the term NAFLD, or fatty liver, reporting that the words used within the acronym “MASLD” were difficult to understand [51].

Avoidance of social situations due to fatigue, perceived stigma, or other anxieties is an issue of concern due to the potential of these factors resulting in social isolation or loneliness and the possible implications of such. The World Health Organization draws attention to the importance of social connections with regard to both mental and physical health, reporting that social isolation or loneliness can affect health in a similar manner to established risk factors including obesity and smoking [52]. In addition, such concepts may prevent the individual from seeking health information and treatment [9].

However, despite the issues raised above regarding avoidance of social situations and perceived stigma, it was also interesting to observe the positivity and proactivity that some participants expressed. This was primarily reported as maintaining a positive attitude, actively seeking information, or participating in support groups of the British Liver Trust. Furthermore, one participant mentioned participating in “fun runs” to raise awareness of liver disease, and another explained involvement in a community project to campaign and enhance awareness of the condition.

### 4.3. Future Concerns and Support Needs

Frustration, fear, and anger also represent key emotions regarding a lack of follow-up after the initial diagnosis, whereby participants naturally felt a need to learn if their condition was worsening or improving. Understandably, participants were concerned regarding the future, especially with regard to possible progression of MASLD. This was particularly evident through discussions with the majority of participants who were not receiving any follow-up. Participants were frustrated by the lack of information and follow-up and wanted to know if there was anything further they should be doing and whether the disease was improving or worsening over time. Disappointment regarding the lack of concise information or oversimplified guidance, together with fears of disease progression, is consistent with reports from previous studies [12,16]. Thus, it is important to recognize factors such as health literacy and the implications with regard to lifestyle modification and HCP–patient communication [14].

Some participants had further concerns owing to a known family history of liver disease and reflected on the loss of a parent as a result of this, questioning whether there may be a genetic factor involved in their own diagnosis. Participants expressed a desire for further information, reassurance, and advice on lifestyle changes relevant to their own circumstances. Furthermore, they raised the importance of receiving regular monitoring of their condition and potential referral to a specialist or a dietician.

## 5. Summary of Identified Themes/Sub-Themes and Implications for Future Research and Relevant Action Plans in the Context of MASLD Care Pathways

Table 3 summarizes the four themes and the corresponding sub-themes that were identified from the interviews of the study participants, including one representative quote for each sub-theme.

Overall, the findings of the current study indicate that clear and comprehensive information should be provided to patients with MASLD at the point of diagnosis and that the concept of lifestyle modification should be sufficiently explained early in the management plan, taking into account personal circumstances/needs and potential barriers. To that aim, a multi-disciplinary management approach/plan from the time of diagnosis may also help to overcome communication issues and better support needs such as those reported by our study participants. To what extent and which specific multi-disciplinary approaches to MASLD management can significantly improve the emotional well-being of patients, patient-reported outcomes, and patient satisfaction in the context of MASLD care pathways should be further explored by studies that would follow these patients from the point of diagnosis.

In addition, the issues/themes identified by the present study could potentially guide the development of a relevant patient checklist for use by HCPs when communicating the initial MASLD diagnosis. Providing essential and patient-friendly information or signposting to reliable and easily accessible patient-oriented resources for MASLD through such a standardized checklist approach could also potentially help to address communication issues at the time of diagnosis and related psychological distress. The emotional distress reported by participants when receiving the MASLD diagnosis may also be reduced by adopting an empathetic approach and/or referral to compassion-based initiatives, such as motivational interviewing or referral to social prescribers, where such resources are available. The impact and exact role of social prescribers or other support initiatives that could be embedded in MASLD care pathways to better support the patients also merit targeted research.

Finally, taking as an example that some participants in the current study relayed their own suggestions regarding the future care of patients with MASLD, further research could also be directed towards learning directly from suggestions by the patients regarding the co-creation of relevant interventions/initiatives which could be embedded in MASLD care pathways. Similarly, it could prove beneficial to conduct qualitative studies among HCPs involved in MASLD care to gain a deeper insight into their perspectives regarding the diagnosis and care of individuals with MASLD.

## 6. Strengths and Limitations

This qualitative study supports the findings from previous studies regarding the issues faced by adults with MASLD. Indeed, this study enhances previous work by providing a broad picture of the individuals’ journey from the point of diagnosis through to future concerns and support needs. However, certain study limitations should be acknowledged. Firstly, the MASLD/NAFLD diagnosis and other relevant data (e.g., weekly alcohol intake) were self-reported by the participants, without access to medical records. Thus, in the context of this qualitative study, it was not possible to capture precise clinical data and ascertain with certainty the exact alcohol intake of participants in order to define MASLD severity and also definitely rule out the possibility of metabolic dysfunction and alcohol-associated liver disease (MetALD) or alcoholic liver disease (ALD) in the study sample.

Moreover, as this is a UK-based study, all study participants were from the UK, with the majority belonging to a British white ethnic group. Thus, we were unable to fully capture an ethnically diverse group of participants, and the present findings might not reflect the lived experiences of patients with MASLD from other countries or other ethnic groups. Although it was beyond the scope of this qualitative study to focus on ethnic minority groups, this is an issue of importance that future studies should explore to identify culture/ethnic-related factors that might shape lifestyle, illness/stigma perception, healthcare-seeking behaviors, and disease management across different ethnic groups. Furthermore, as participants were recruited through the website of the British Liver Trust, volunteer bias is a possible limitation of this study that might constrain the transferability of the present findings to the wider MASLD population, and particularly to patients with MASLD who are less likely to seek information through such online resources. Indeed, it is possible that the findings of the present study may actually underestimate the true severity of unmet clinical and support needs within this patient population, particularly for those patients who are less proactive in seeking information and support for their MASLD diagnosis. However, as our participants were individuals who were actively trying to obtain further support and information on MASLD, they potentially represent an appropriate audience for targeted self-management interventions.

In addition, the present qualitative study focused exclusively on the patient perspective. Thus, issues highlighted by the reported lived experience of participants regarding poor communication/information and lack of follow-up that could be due to potential knowledge gaps among HPCs should be interpreted with caution and require targeted research to be thoroughly investigated.

It should also be noted that certain limitations apply to all studies that are qualitative in nature. Such limitations may include the following: the inability of qualitative studies to identify causal relationships; issues regarding generalizability/external validity; and issues such as researcher subjectivity/bias. As such inferences regarding screening of high-risk MASLD groups, MASLD professional training, and MASLD care pathways should be viewed as exploratory implications based on the reported lived experiences of the study participants rather than direct conclusions. Furthermore, this qualitative study does not evaluate the impact and clinical outcomes of structured MASLD care pathways or the effectiveness of interventions for MASLD. Finally, it is likely that when taking part in a qualitative study, participants may be more outspoken and passionate regarding their experiences of living with the condition under investigation.

## 7. Conclusions

The findings of the present qualitative study highlight a number of key issues that could impede the optimal management of MASLD. Many of the participants in our study had received the diagnosis incidentally, resulting in emotions such as shock and confusion. Participants raised a number of issues including lack of clarity and information at the point of diagnosis, concerns regarding information obtainable via the internet, and factors related to lifestyle modification, symptoms, social relationships, and stigma. Future concerns related mainly to fears of disease progression, while support needs were predominantly focused on information and follow-up. The results from this research support the findings of other relevant studies [10,12,16,19] and further clarify emerging issues of concern in relation to the lived experiences of adults with a diagnosis of MASLD.

Our study supports and enhances research within this field, revealing the emotional journey from the point of diagnosis, confusion and frustration relating to lack of information, and the need for more individualized advice relating to lifestyle modification.

Given the individual personal circumstances of adults with MASLD and the growing burden of the disease, it would seem imperative that a holistic care pathway is in place to support these individuals. Of interest, some participants made suggestions themselves regarding future approaches to the care of patients with MASLD, including a specific care pathway, signposting to bona fide resources such as the British Liver Trust, raising awareness, and investing in research. One participant further noted that the findings from our study could be utilized as a checklist for clinicians, to aid them in understanding the concerns and needs of patients. This highlights that the voice of patients should be taken into account in the context of a more patient-centered approach for the management of MASLD.

Of note, based on the design of the present study, the recruited study sample is representative of individuals who are more likely to seek further information and support regarding MASLD through online resources such as those offered by the British Liver Trust. Therefore, it is rather alarming that issues regarding adequate communication and information for MASLD are highlighted by those who actively seek support and educational resources on MASLD. This further suggests that those who are less proactive or belong to particularly vulnerable groups are potentially more likely to encounter such problems and may have even more pronounced unmet support needs.

Due to the complex and challenging nature of MASLD, a holistic approach incorporating compassion and understanding could help to improve both the mental and physical health of patients with MASLD. Overall, the findings from this study contribute to, and enhance the evidence in relation to the needs of adults with MASLD and can help to inform improvements to self-management, targeted holistic support, and appropriate care pathways for these patients.

## Figures and Tables

**Table 1:** Topic/question guide for the interviews of the present qualitative study.

Example Questions from Topic/Question Guide:
How would you describe your health in general, both now and in the past?
Can you tell me something about your NAFLD/MASLD diagnosis?
Had you come across this condition before diagnosis?
What were your thoughts and feelings when you were diagnosed?
What information were you given? What support was offered?
Have you been able to find support and information since diagnosis?
How easy was it for you to find information…?
Did you share the diagnosis with family, friends and colleagues?
Are you receiving follow-up treatment or help with managing the condition?
Do you experience physical symptoms that you believe are related to your condition?
On the less physical side of things, do you suffer from any psychological issues that you believe are related to your condition?
To what extent does the condition affect your social relationships?
What additional support do you think will be helpful to you at the current time?
Is there anything else that you would like to add?

**Table 2:** Selected pertinent demographic and clinical characteristics that were reported by the study participants.

Participant Demographic/Clinical Characteristics		N
Gender	Male	10
	Female	15
Age	<40 years	2
	41–50 years	4
	51–60 years	8
	61–70 years	9
	>70 years	2
Occupation	Full-time employed	10
	Part-time employed	2
	Unemployed	1
	Retired	10
	House person	1
	Other	1
Education	Secondary	9
	College	3
	Undergraduate	2
	Postgraduate	8
	Doctorate	2
	Other not specified	1
Ethnic Background/Group	White	22
	Other	3
NAFLD/MASLD Diagnosis	Liver ultrasound/Fibroscan	16
	Liver ultrasound/Fibroscan and liver CT	5
	Liver ultrasound/Fibroscan and liver MRI	2
	Liver ultrasound/Fibroscan and liver CT and MRI	1
	Liver biopsy	0
	Other not specified	1
Other health conditions	Yes	21
	Not aware/don’t know	4
Body mass index (BMI)	<25 kg/m^2^	5
	25–30 kg/m^2^	3
	>30 kg/m^2^	17
Alcohol intake (self-reported units per week)	None	17
	Rarely/socially/special occasions	2
	Three	1
	Four	1
	Six	2
	Fourteen	2

NAFLD, non-alcoholic fatty liver disease; MASLD, metabolic dysfunction-associated steatotic liver disease; CT, computed tomography; MRI, magnetic resonance imaging.

**Table 3:** The themes/sub-themes that were identified from the interviews of the study participants, including one representative quote per sub-theme.

Theme	Theme Description	Sub-Theme	Example Quote for Each Sub-Theme
**1. Experience of diagnosis**	Focuses on participants’ experiences both at the point of and beyond the initial MASLD diagnosis.	**1a**. Communication at point of diagnosis ^#^	*“…they kind of didn’t really tell me what they’d seen until much later and then told me oh yes, fatty liver…”*
		**1b**. Emotions at point of diagnosis ^#^	*“I was very scared, and I was unable to sleep at night…”*
		**1c**. Follow-up/disease progression ^#^	*“…I did say so, what’s the plan now? And I was told, well, there’s nothing…”*
**2. Independently seeking further information**	Addresses the methods utilized by participants in order to obtain further information.	**2a**. Internet searches ^#^	*“…but that’s it…other than Googling it…you hope for the best and you read the worst…”*
		**2b**. British Liver Trust ^#^	*“…I then came across the BLT and I phoned their helpline and the nurses were great. They helped me put things into perspective…”*
**3. Living with MASLD**	Explores participants’ lived experiences post-MASLD diagnosis.	**3a**. Lifestyle modification ^#^	*“…They just said you’ll have to diet…I know that already, but I want a bit of help…”*
		**3b**. Physical and psychological problems ^# #^	*“… I’m wiped out completely. I wake up tired. I could fall asleep now, you know. It’s just…it’s complete fatigue…”*
		**3c**. Social relationships/stigma ^# #^	*“…people just look at you and think you’re an alcoholic or whatever…”*
		**3d**. Positivity and proactivity ^# # #^	*“…I do fun runs……we have a lovely community of people that have had liver transplants or going through things and they start to kind of help like promote the positive things which I think is good…”*
**4. Thoughts regarding the future**	This theme explores thoughts and concerns regarding the future, together with support needs.	**4a**. Concerns—disease progression ^# #^	“…its *a ticking time bomb if there aren’t options to do something about it…”*
		**4b**. Family history of liver disease ^# # #^	*“…my mum died of liver cancer…It is disappointing, you know, especially when I found out that I should have been reviewed yearly, and I’ve not been…”*
		**4c**. Support needed ^# #^	*“…I don’t understand why they put these tests results on the NHS app where the patient can see them, and yet they do not follow up on this…I could not see my GP…”*

Information richness/power key for each sub-theme: ^#^: the subtheme was discussed by the majority of the study participants; ^# #^: the subtheme was discussed by many study participants; ^# # #^: the subtheme was discussed by some study participants.

## Data Availability

Data are available via the corresponding authors upon reasonable request and where it is ethically acceptable and does not violate the protection of study participants or other valid ethical, privacy, or security concerns.

## Supplementary Materials

The following supporting information can be downloaded at https://www.mdpi.com/article/10.3390/healthcare14162569/s1: Supplementary Table S1. Reporting of the present qualitative study according to the 21-item checklist of the Standards for Reporting Qualitative Research (SRQR) [28].

## Abbreviations

The following abbreviations are used in this manuscript:

CVD	Cardiovascular disease
GP	General practitioner
HCP	Healthcare professional
MASLD	Metabolic dysfunction-associated steatotic liver disease
NAFLD	Non-alcoholic fatty liver disease

## References

[1] Younossi Z.M., Kalligeros M., Henry L. (2025). Epidemiology of metabolic dysfunction-associated steatotic liver disease. Clin. Mol. Hepatol..

[2] Kyrou I., Randeva H.S., Tsigos C., Kaltsas G., Weickert M.O., Feingold K.R., Anawalt B., Boyce A., Chrousos G., de Herder W.W., Dhatariya K., Dungan J.M., Hershman J., Hofland S., Kalra G. (2018). Clinical Problems Caused by Obesity.

[3] Godoy-Matos A.F., Silva Junior W.S., Valerio C.M. (2020). NAFLD as a continuum: From obesity to metabolic syndrome and diabetes. Diabetol. Metab. Syndr..

[4] Paternostro R., Trauner M. (2022). Current treatment of non-alcoholic fatty liver disease. J. Intern. Med..

[5] Shea S., Lionis C., Kite C., Lagojda L., Uthman O.A., Dallaway A., Atkinson L., Chaggar S.S., Randeva H.S., Kyrou I. (2024). Non-alcoholic fatty liver disease and coexisting depression, anxiety and/or stress in adults: A systematic review and meta-analysis. Front. Endocrinol..

[6] Hagstrom H., Shang Y., Hegmar H., Nasr P. (2024). Natural history and progression of metabolic dysfunction-associated steatotic liver disease. Lancet Gastroenterol. Hepatol..

[7] Shang Y., Widman L., Zhang X., Fernandes G., Melaragno M.G., Engel S.S., Vessby J., Ekstedt M., Hagström H. (2026). Characteristics and Clinical Outcomes in Patients With Cirrhosis due to MASLD in Sweden. Liver Int..

[8] Shea S., Lionis C., Atkinson L., Kite C., Lagojda L., Chaggar S.S., Kyrou I., Randeva H.S. (2023). Support Needs and Coping Strategies in Non-Alcoholic Fatty Liver Disease (NAFLD): A Multidisciplinary Approach to Potential Unmet Challenges Beyond Pharmacological Treatment. Livers.

[9] Shea S., Lionis C., Kite C., Atkinson L., Lagojda L., Chaggar S.S., Kyrou I., Randeva H.S. (2023). Challenges in the Management of Non-Alcoholic Fatty Liver Disease (NAFLD): Towards a Compassionate Approach. Livers.

[10] Deshpande K., Olynyk J., Ayonrinde O., Kazunori N. (2025). Exercise Perceptions in People with Metabolic Dysfunction-Associated Steatotic Liver Disease: Insights from a Qualitative Study. J. Endocrinol. Metab..

[11] Alemany-Pages M., Moura-Ramos M., Araujo S., Macedo M.P., Ribeiro R.T., do O.D., Ramalho-Santos J., Azul A.M. (2020). Insights from qualitative research on NAFLD awareness with a cohort of T2DM patients: Time to go public with insulin resistance?. BMC Public Health.

[12] Tincopa M.A., Wong J., Fetters M., Lok A.S. (2021). Patient disease knowledge, attitudes and behaviours related to non-alcoholic fatty liver disease: A qualitative study. BMJ Open Gastroenterol..

[13] Wieland A.C., Mettler P., McDermott M.T., Crane L.A., Cicutto L.C., Bambha K.M. (2015). Low awareness of nonalcoholic fatty liver disease among patients at high metabolic risk. J. Clin. Gastroenterol..

[14] Gergianaki I., Anastasiou F., Papadakis S., Anastasaki M., Linardakis M., Mendive J., Heyens L.J.M., Koek G., Muris J., Lionis C. (2026). Towards a Better Understanding of MASLD: Patient Health Literacy, Illness Perception, and Awareness. Diseases.

[15] Shea S., Lionis C., Kite C., Atkinson L., Chaggar S.S., Randeva H.S., Kyrou I. (2021). Non-Alcoholic Fatty Liver Disease (NAFLD) and Potential Links to Depression, Anxiety, and Chronic Stress. Biomedicines.

[16] Jensen-LeBlanc J.L., Andreassen P., Lauridsen M.M., Gronkjaer L.L. (2026). Patients’ Experience of Stigma as the Hidden Burden of Metabolic Dysfunction-Associated Steatotic Liver Disease: A Descriptive Qualitative Study with Thematic Analysis. Healthcare.

[17] Health Research Authority Patient Preferences for NAFLD Care. https://www.hra.nhs.uk/planning-and-improving-research/application-summaries/research-summaries/patient-preferences-for-nafld-care/.

[18] Boeckmans J., Hagstrom H., Cryer D.R., Schattenberg J.M. (2025). The importance of patient engagement in the multimodal treatment of MASLD. Commun. Med..

[19] Stine J.G., Medic N., Pettersson B., Venerus M., Blau J.E. (2024). The health care experience of adults with metabolic dysfunction-associated steatohepatitis and influence of PNPLA3: A qualitative study. Hepatol. Commun..

[20] Vasileiou K., Barnett J., Thorpe S., Young T. (2018). Characterising and justifying sample size sufficiency in interview-based studies: Systematic analysis of qualitative health research over a 15-year period. BMC Med. Res. Methodol..

[21] Hennink M., Kaiser B.N. (2022). Sample sizes for saturation in qualitative research: A systematic review of empirical tests. Soc. Sci. Med..

[22] Carrillo A., Feig E.H., Harnedy L.E., Huffman J.C., Park E.R., Thorndike A.N., Kim S., A Millstein R. (2022). The role of positive psychological constructs in diet and eating behavior among people with metabolic syndrome: A qualitative study. Health Psychol. Open.

[23] Imhagen A., Karlsson J., Jansson S., Anderzen-Carlsson A. (2023). A lifelong struggle for a lighter tomorrow: A qualitative study on experiences of obesity in primary healthcare patients. J. Clin. Nurs..

[24] Martinez Leal I., Pillai A.B., Foreman J.T., Siu K.W., Heredia N.I., Escalante C.P., Manzullo E.F., Christie A.J., Lacourt T.E., Razouki Z.A. (2024). A Qualitative Study of the Knowledge of Metabolic Syndrome, Attitudes about Lifestyle Modifications, and Preferences for Lifestyle Interventions among Patients with Cancer and Metabolic Syndrome. Cancers.

[25] Peng X., Guo X., Li H., Wang D., Liu C., Du Y. (2022). A Qualitative Exploration of Self-Management Behaviors and Influencing Factors in Patients With Type 2 Diabetes. Front. Endocrinol..

[26] Braun V., Clarke V. (2021). Thematic Analysis: A Practical Guide.

[27] Malterud K., Siersma V.D., Guassora A.D. (2016). Sample Size in Qualitative Interview Studies: Guided by Information Power. Qual. Health Res..

[28] O’Brien B.C., Harris I.B., Beckman T.J., Reed D.A., Cook D.A. (2014). Standards for reporting qualitative research: A synthesis of recommendations. Acad. Med..

[29] Clarke V., Braun V. (2017). Thematic Analysis. J. Posit. Psychol..

[30] Braun V., Clarke V. (2006). Using thematic analysis in psychology. Qual. Res. Psychol..

[31] Czapla B.C., Dalvi A., Hu J., Moran I.J., Wijarnpreecha K., Chen V.L. (2025). Physical activity, diet, and social determinants of health associate with health related quality of life and fibrosis in MASLD. Sci. Rep..

[32] Golabi P., Otgonsuren M., Cable R., Felix S., Koenig A., Sayiner M., Younossi Z.M. (2016). Non-alcoholic Fatty Liver Disease (NAFLD) is associated with impairment of Health Related Quality of Life (HRQOL). Health Qual. Life Outcomes.

[33] Sapmaz A., Paik A., Henry L. (2024). A comprehensive review of patient-reported outcomes in metabolic dysfunction-associated steatotic liver disease. Metab. Target Organ Damage.

[34] Dokmak A., Lizaola-Mayo B., Trivedi H.D. (2021). The Impact of Nonalcoholic Fatty Liver Disease in Primary Care: A Population Health Perspective. Am. J. Med..

[35] Islam K.B., Brandman D., Chu J.N., Goldman M.L., Fox R.K. (2023). Primary Care Providers and Nonalcoholic Fatty Liver Disease: A Needs Assessment Survey. Dig. Dis. Sci..

[36] Castera L., Alazawi W., Bugianesi E., Caussy C., Federici M., Romero-Gomez M., Schattenberg J.M., Basuroy R., Prasad P., Estulin D. (2025). A European Survey to Identify Challenges in the Management of Metabolic Dysfunction-Associated Steatotic Liver Disease. Liver Int..

[37] Zhu K., Jogendran M., Zhang Y., Hussaini T., Chahal D., Yoshida E.M. (2025). Cross-sectional Study of Resident Physician Knowledge and Perceptions Regarding MASLD in Canada. Can. Liver J..

[38] Lionis C., Papadakis S., Anastasaki M., Aligizakis E., Anastasiou F., Francque S., Gergianaki I., Mendive J.M., Marketou M., Muris J. (2024). Practice Recommendations for the Management of MASLD in Primary Care: Consensus Results. Diseases.

[39] Atherton H., Leach H., Mortell R., Parsons J. (2025). What patients want from access to UK general practice: Systematic review. Br. J. Gen. Pract..

[40] BMJ (2026). GP deserts: Data show chronic shortage of family doctors in England. BMJ.

[41] Kanwal F., Shubrook J.H., Adams L.A., Pfotenhauer K., Wai-Sun Wong V., Wright E., Abdelmalek M.F., Harrison S.A., Loomba R., Mantzoros C.S. (2021). Clinical Care Pathway for the Risk Stratification and Management of Patients with Nonalcoholic Fatty Liver Disease. Gastroenterology.

[42] Jarvis H., Worsfold J., Hebditch V., Ryder S. (2021). Engagement with community liver disease management across the UK: A cross-sectional survey. BJGP Open.

[43] RGCP Digital Harms are a Modern Determinant of Health—And a Population Health Issue 2026. https://www.rcgp.org.uk/News/digital-harms-population-health.

[44] Pedersen M.R.L., Hansen A.F., Elmose-Osterlund K. (2021). Motives and Barriers Related to Physical Activity and Sport across Social Backgrounds: Implications for Health Promotion. Int. J. Environ. Res. Public Health.

[45] Francque S.M., Marchesini G., Kautz A., Walmsley M., Dorner R., Lazarus J.V., Zelber-Sagi S., Hallsworth K., Busetto L., Fruhbeck G. (2021). Non-alcoholic fatty liver disease: A patient guideline. JHEP Rep..

[46] Berry P., Kotha S. (2022). The Challenging Ethical Landscape of Non-alcoholic Fatty Liver Disease. EMJ Hepatol..

[47] Newton J.L., Jones D.E., Henderson E., Kane L., Wilton K., Burt A.D., Day C.P. (2008). Fatigue in non-alcoholic fatty liver disease (NAFLD) is significant and associates with inactivity and excessive daytime sleepiness but not with liver disease severity or insuling resistance. Gut.

[48] Younossi Z., Yilmaz Y., Yu M.L., El-Kassas M., Fernández M.I.C., Eguchi Y., Papatheodoridis G., Wong V.W.-S., Duseja A.K., Singal A.K. (2025). FRI-030 In patients with metabolic dysfunction-associated steatotic liver disease, sleep disturbance is highly prevalent and associated with a profound impairment of health-related quality of life. J. Hepatol..

[49] Carol M., Perez-Guasch M., Sola E., Cervera M., Martinez S., Juanola A., Ma A.T., Avitabile E., Napoleone L., Pose E. (2022). Stigmatization is common in patients with non-alcoholic fatty liver disease and correlates with quality of life. PLoS ONE.

[50] Rinella M.E., Lazarus J.V., Ratziu V., Francque S.M., Sanyal A.J., Kanwal F., Terrault N.A., Rinella M.E. (2023). Reply: A multi-society Delphi consensus statement on new fatty liver disease nomenclature. J. Hepatol..

[51] Williams K., Cuthbertson D.J., Roper L., Rowe I., Hydes T.J. (2025). Qualitative study explores patient preferences for metabolic dysfunction-associated steatotic liver disease nomenclature. J. Hepatol..

[52] WHO (2022). Social Isolation and Loneliness. https://www.who.int/teams/social-determinants-of-health/demographic-change-and-healthy-ageing/social-isolation-and-loneliness.

