# Supplementary material for: Lived Experience, Concerns, and Support Needs of Adults with Metabolic Dysfunction-Associated Steatotic Liver Disease (MASLD): A Qualitative Study
Source: Healthcare (Basel). 2026 Aug 17;14(16):2569. doi: 10.3390/healthcare14162569 (PMC13512164; doi:10.3390/healthcare14162569)
Supplement: Supplementary file 1 [file healthcare-14-02569-s001.zip › healthcare-4425130-supplementary.pdf]

Supplementary Table S1. Reporting of the present qualitative study according to the 21-item checklist of the Standards for Reporting Qualitative Research (SRQR) [28]

SPQR ITEM	Page/Line Number(s)
**Title and abstract (Items 1-2)**	
1. Title	Page 1, lines 2-4
2. Abstract	Page 2, lines 48-73
**Introduction (Items 3-4)**	
3. Problem formulation	Pages 2-3, lines 78-124
4. Purpose or research question	Page 3, lines 125-139
**Methods (Items 5-15)**	
5. Qualitative approach and research paradigm	Page 4, lines 145-154<br>Page 5, lines 242-243
6. Researcher characteristics and reflexivity	Pages 5-6, lines 242-247; and page 6, lines 267-268
7. Context	Page 4, lines 147-154, and 160-181; page 5-6, lines 242-247; page 6, lines 249-250
8. Sampling strategy	Page 4, lines 140-154, and lines 157-159
9. Ethical issues pertaining to human subjects	Page 6, lines 273-275
10. Data collection methods	Page 4, lines 171-181
11. Data collection instruments and technologies	Page 4, lines 171-181<br>Page 6, lines 246-247
12. Units of study (Number and relevant characteristics of participants, etc.)	Page 4, lines 142-154 and 157-159
13. Data processing	Page 4, lines 172-189, and page 6, lines 249-266
14. Data analysis	Page 6, lines 242-266
15. Techniques to enhance trustworthiness	Pages 4-5, lines 179-235
**Results/findings (Items 16-17)**	
16. Synthesis and interpretation	Pages 6-16, lines 278-688
17. Links to empirical data	Pages 8-16, lines 299-688
**Discussion (Items 18-19)**	
18. Integration with prior work, implications, transferability, and contribution(s) to the field	Pages 16-23, lines 690-943
19. Limitations	Pages 23-24, lines 947-991
**Other (Items 20-21)**	
20. Conflicts of interest	Pages 25, line 1057<br>(No conflict of interest)
21. Funding	Pages 25, line 1044<br>(No external funding)

Reference for Supplementary Table S1:

O'Brien BC, Harris IB, Beckman TJ, Reed DA, Cook DA. Standards for reporting qualitative research: a synthesis of recommendations. Acad Med. 2014;89(9):1245-51.
